# Correction: Design, synthesis, pharmacological evaluation, and *in silico* studies of the activity of novel spiro pyrrolo[3,4-*d*]pyrimidine derivatives

**DOI:** 10.1039/d4ra90128b

**Published:** 2024-11-13

**Authors:** Abdullah Y. A. Alzahrani, Wesam S. Shehab, Asmaa H. Amer, Mohamed G. Assy, Samar M. Mouneir, Maged Abdelaziz, Atef M. Abdel Hamid

**Affiliations:** a Department of Chemistry, Faculty of Science and Arts, King Khalid University Mohail Assir Saudi Arabia ayalzahrani@kku.edu.sa; b Department of Chemistry, Faculty of Science, Zagazig University Zagazig 44519 Egypt dr.wesamshehab@gmail.com wsshehab@zu.edu.eg; c Department of Pharmacology, Faculty of Veterinary Medicine, Cairo University Cairo 12211 Egypt

## Abstract

Correction for ‘Design, synthesis, pharmacological evaluation, and *in silico* studies of the activity of novel spiro pyrrolo[3,4-*d*]pyrimidine derivatives’ by Abdullah Y. A. Alzahrani *et al.*, *RSC Adv.*, 2024, **14**, 995–1008, https://doi.org/10.1039/D3RA07078F.

The authors regret that the name of one of the authors (Maged Abdelaziz) was shown incorrectly in the original article. The original article has been updated and the corrected author list is as shown above.

The Royal Society of Chemistry apologises for these errors and any consequent inconvenience to authors and readers.

